# Supplementary material for: Charging modulation of the pyridine nitrogen of covalent organic frameworks for promoting oxygen reduction reaction
Source: Nat Commun. 2024 Feb 29;15:1889. doi: 10.1038/s41467-024-46291-y (PMC10904383; doi:10.1038/s41467-024-46291-y)
Supplement: Supplementary file 1 — Supplementary Information [file 41467_2024_46291_MOESM1_ESM.pdf]

Supporting Information

Charging modulation of the pyridine nitrogen of covalent organic frameworks for promoting oxygen reduction reaction

Xiubei Yang, Qizheng An, Xuewen Li, Yubin Fu, * *Shuai Yang, Minghao Liu, Qing Xu,* * *Gaofeng Zeng* *

Characterization. ^{1}H NMR spectra were measured on a Bruker 400 MHz spectrometer, while chemical shifts (δ in ppm) were determined using a standard of the solvent residual proton. Fourier transform infrared (FT-IR) spectra were recorded on a JASCO model FT IR-6100 infrared spectrometer. X-ray diffraction (XRD) data were recorded on a Bruker D8 Focus Powder X-ray Diffractometer by using powder on glass substrate, from $2\theta = 3°$ up to 30° with 0.01° increment. Elemental analysis was performed on an Elementar vario MICRO cube elemental analyzer. TGA measurements were performed on a Discovery TGA under N_2, by heating from 30 to 800 °C at a rate of 10 °C min^{-1}. Nitrogen sorption isotherms were measured at 77 K with a TriStar II instrument (Micromeritics). The Brunauer-Emmett-Teller (BET) method was utilized to calculate the specific surface areas. By using the non-local density functional theory (NLDFT) model, the pore volume was derived from the sorption curve. Morphology images were characterized with a Zeiss Merlin Compact filed emission scanning electron microscope (FE-SEM) equipped with an energy-dispersive X-ray spectroscopy (EDS) system at an electric voltage of 5 KV. HRTEM images were obtained with a transmission electron microscope (TEM, FEI Tecnai G2) installed with an energy dispersive spectrometer (EDS, Oxford). Solid-state Ultraviolet-visible absorption spectra of solution samples were collected using an Agilent Cary 100 UV/Vis spectrophotometer with background correction.

Synthetic Procedures. 4,4',4'',4'''- (pyrene-1,3,6,8-tetrayl)-tetraaniline (PY), [2,2'-bipyridine] -5,5'-dicarbaldehyde (BPY) and paraformaldehyde were purchased from Jilin Chinese Academy of Sciences-Yanshen Technology Co., Ltd. The Mesitylene, *n*-Butanol, 1,2-dichlorobenzene, methyl alcohol (MeOH), acetonitrile and acetone Tetrahydrofuran (THF) were purchased from Aladdin Chemicals. All the other solvents were purchased from Aladdin Chemicals and used as received without further purification. All the chemical materials used were from commercial suppliers without further purification.

Electrochemical measurements. On the basis of ring and disk currents, the electron-transfer number (n) and four-electron selectivity of catalysts based on the H_2O_2 yield [H_2O_2 (%)] were

calculated from the equations of $n = 4\, I_D/[(\frac{I_R}{N}) + I_D]$ and $H2O2\,(\%) = 200\,(\frac{I_R}{N})/[(\frac{I_R}{N}) + I_D]$, in which I_R and I_D are the disk and ring currents, respectively, and the ring collection efficiency N is 0.37. The Tafel slope was estimated by linear fitting of the polarization curves according to the Tafel equation ($h = b\ \log j + a$, in which j is the current density and b is the Tafel slope). For the cyclic voltammograms (CV) tests, the potential range was circularly scanned between –0.80 V and 0.10 V at a scan rate of 50 mV s^{-1} after purging O_2 gas for 30 min. For estimation of the double layer capacitance, the electrolyte was deaerated by bubbling with nitrogen, and then the voltammogram was evaluated again in the deaerated electrolyte. The rotating disk electrode (RDE) measurements were conducted at different rotation rates from 400 to 1600 rpm at a scan rate 10 mV s^{-1}.

Turnover frequency (TOF). The turnover frequency (TOF) was evaluated by the following standard equation:

$$TOF = (J\ \times A)/(4\ \times F\ \times n)$$

Where j (A/cm^2) is the current density at a given potential (0.7 V), A is the surface area of the electrode (0.125 cm^2), the number of 4 represents 4 electrons/mol of O_2, F is the Faraday constant (96500 C/mol), and n stands for the number of moles of C atoms in samples.

Computational method: We have employed the VASP[1,2] to perform all the density functional theory (DFT) calculations within the generalized gradient approximation (GGA) using the Perdew-Burke-Ernzerhof (PBE) [3] formulation. We have chosen the projected augmented wave (PAW) potentials [4] to describe the ionic cores. Take valence electrons into account using a plane wave basis set with a kinetic energy cutoff of 450 eV. Partial occupancies of the Kohn-Sham orbitals were allowed using the Gaussian smearing method and a width of 0.05 eV. The electronic energy was considered self-consistent when the energy change was smaller than 10^{-5} eV. A geometry optimization was considered convergent when the energy change was smaller than 0.03 eV/Å. The brillouin zone is sampled with 1 × 1 × 1 Gamma mesh [5].

Gibbs free energy can be obtained by adding corrections including entopic (TS) and zero-point energy (ZPE) to calculated DFT energy, so that $\Delta G = \Delta E_{DFT} + \Delta ZPE - T\Delta S - eU$.

where the E_{DFT} is the calculated DFT reaction energy, ΔZPE is the change in ZPE calculated from the vibrational frequencies and ΔS is the change in the entropy referring to thermodynamics databases. The electrode potential is adopted with respect to the reversible hydrogen electrode, which makes the standard electrochemical potential of electron involved in reaction (G_e) equal to –eU, and the standard electrochemical potential of the proton (G_{H+}) equal to that of the hydrogen atom in gaseous H_2 ($1/2G_{H2}$). Considering that the triplet state of the O_2 molecule is poorly described in the current DFT scheme, the free energy of the O_2 molecule was derived according to $G_{O2} = 2\,G_{H2O} - 2\,G_{H2} + 4.92$.

Supporting Figures:

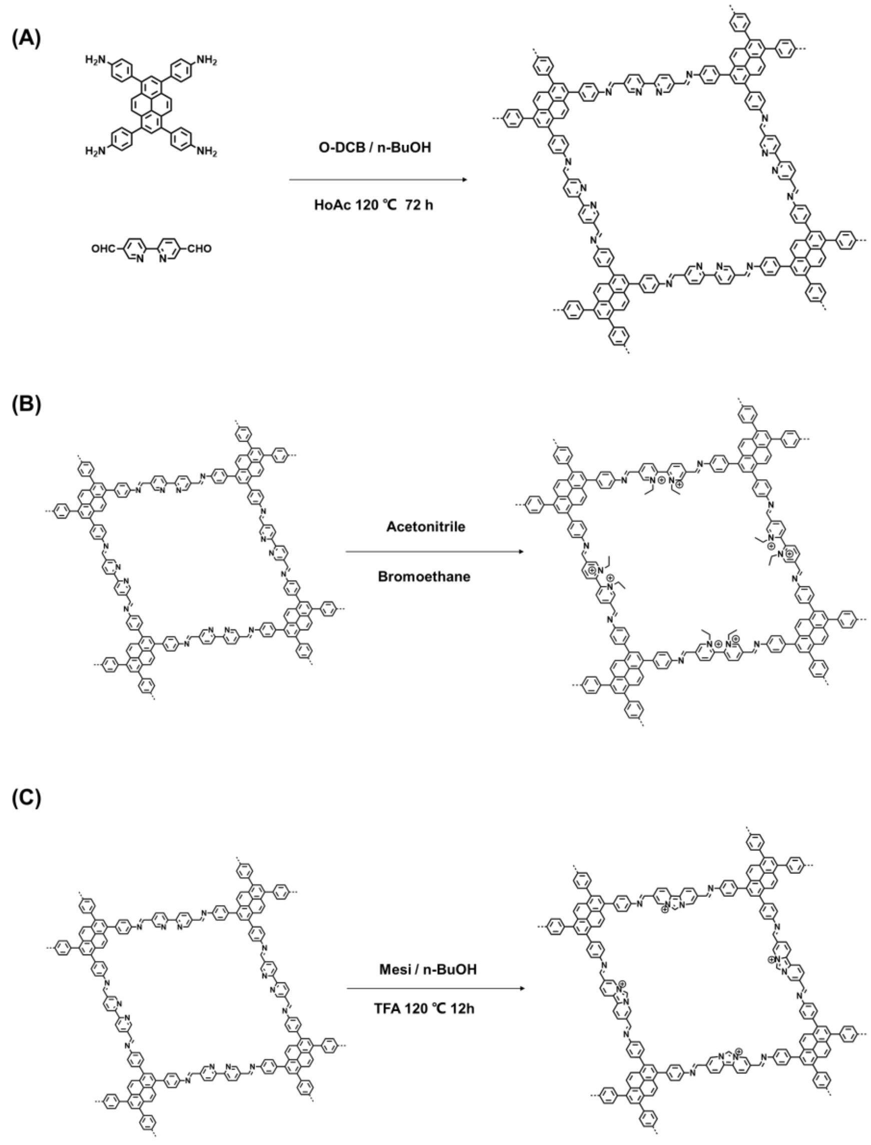

Supplementary Fig. 1 Chemical structures of (A) PY-BPY-COF, (B) *ion*-PY-BPY-COF, and (C) *im*-PY-BPY-COF.

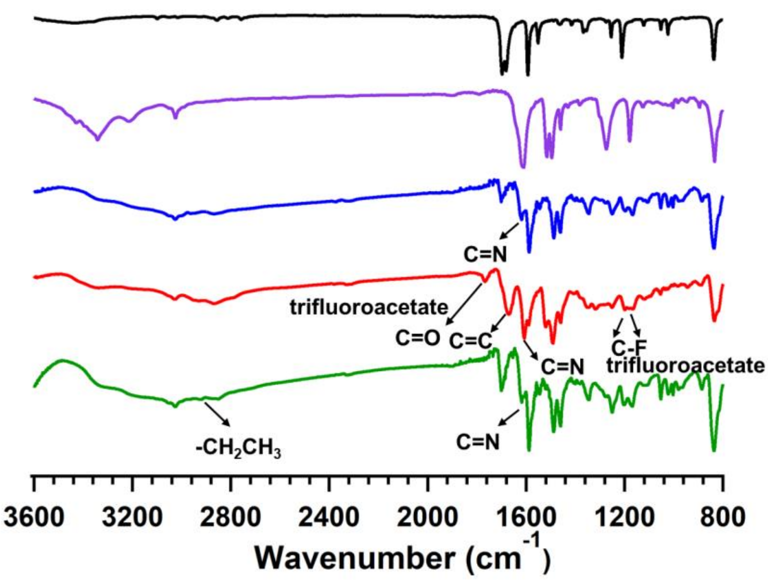

Supplementary Fig. 2 FT-IR spectra of PY-BPY-COF (blue), *ion*-PY-BPY-COF (green), *im*-PY-BPY-COF (red) and the corresponding monomer of PY (black) and BPY (purple).

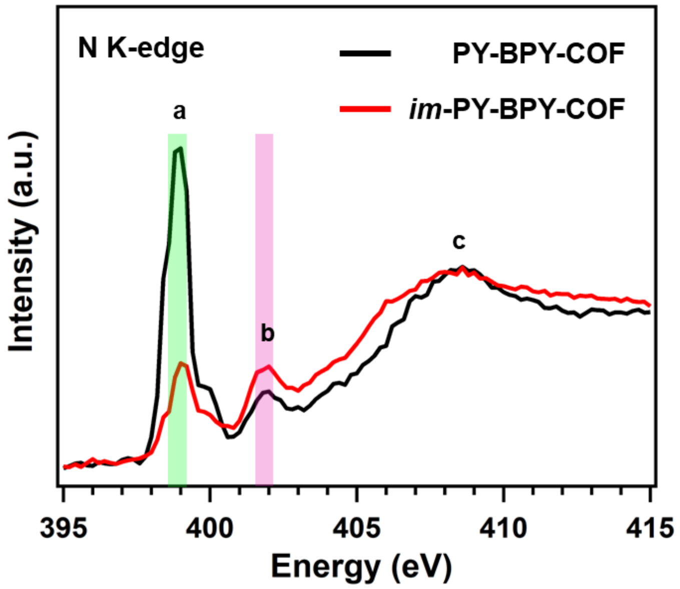

Supplementary Fig. 3 The N K-edge XANES spectra of PY-BPY-COF (black) and *im*-PY-BPY-COF (red).

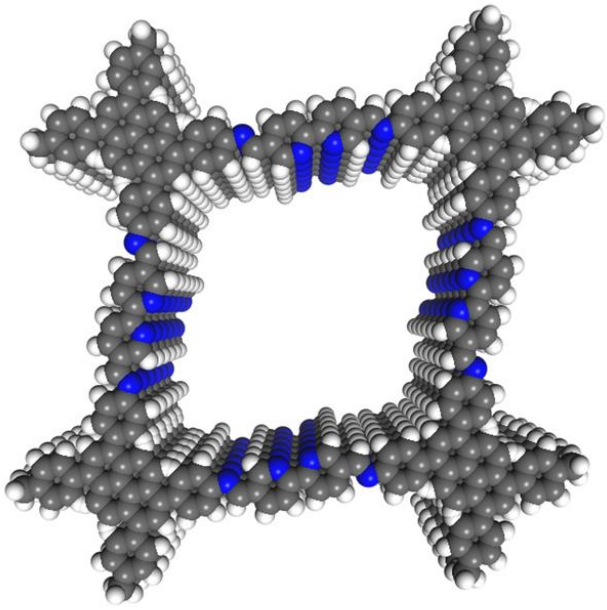

Supplementary Fig. 4 Theoretically modelled eclipsed AA stacking models of PY-BPY-COF.

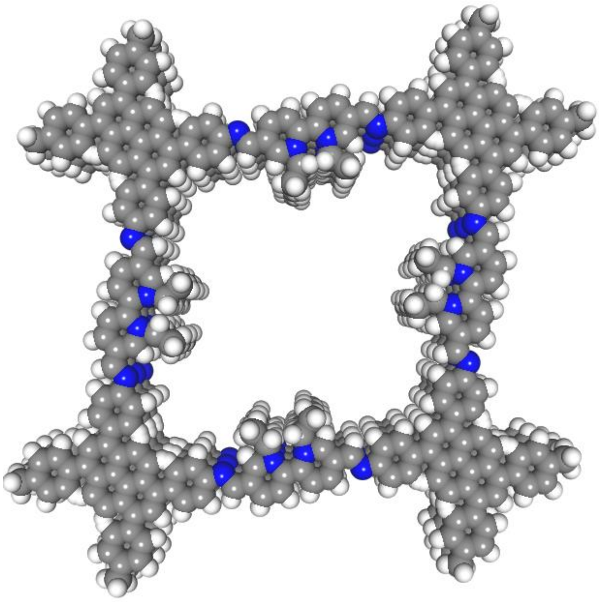

Supplementary Fig. 5 Theoretically modelled eclipsed AA stacking models of *ion*-PY-BPY-COF.

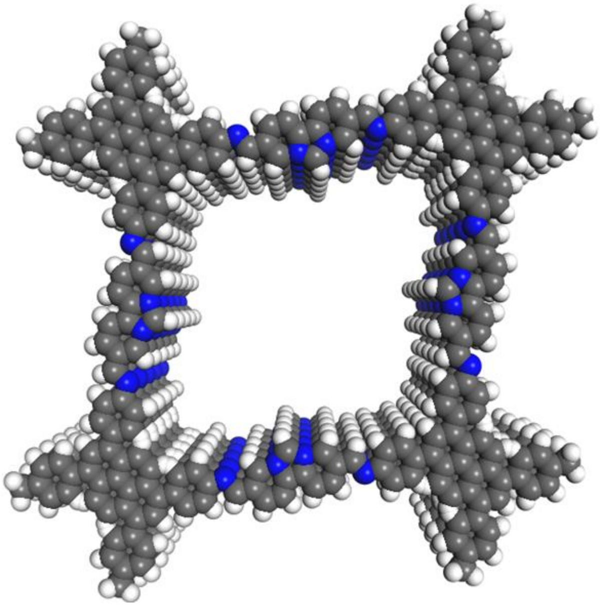

Supplementary Fig. 6 Theoretically modelled eclipsed AA stacking models of *im*-PY-BPY-COF.

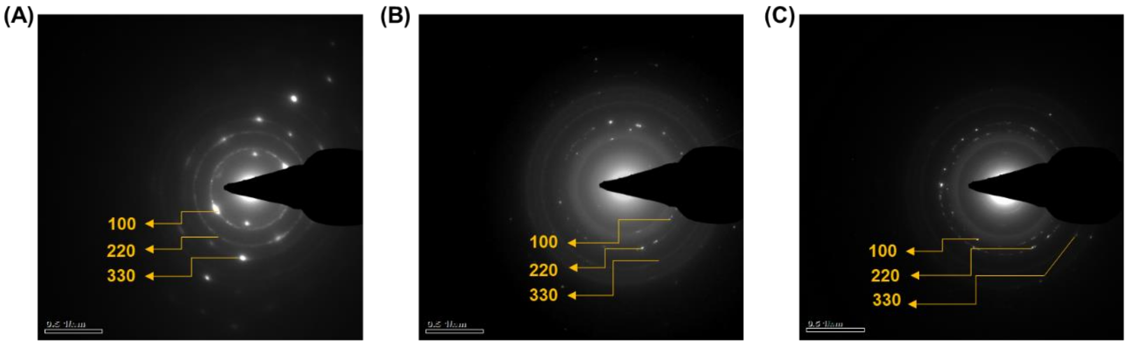

Supplementary Fig. 7 Selected area electron diffraction pattern shown the presence of diffraction rings, corresponding to the crystal planes within the (A) PY-BPY-COF, (B) *ion*-PY-BPY-COF, and (C) *im*-PY-BPY-COF.

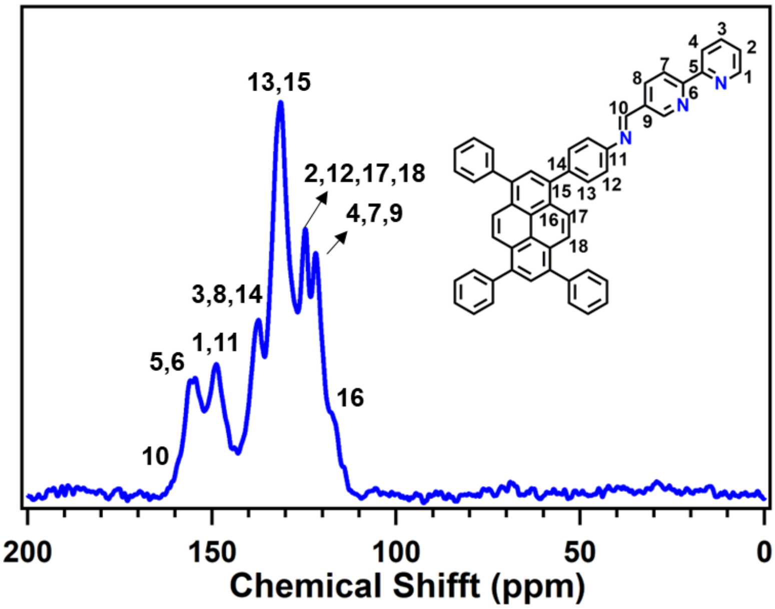

Supplementary Fig. 8 The ^{13}C NMR spectra of PY-BPY-COF.

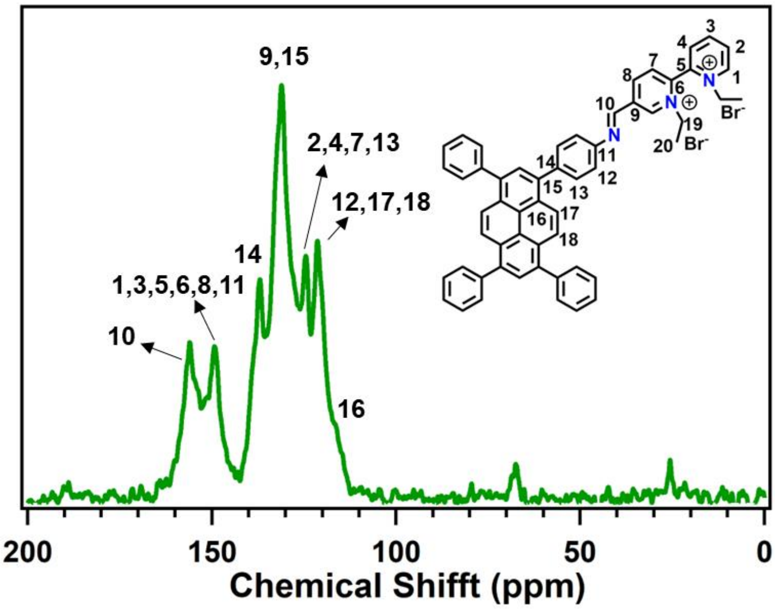

Supplementary Fig. 9 The ^{13}C NMR spectra of *ion*-PY-BPY-COF.

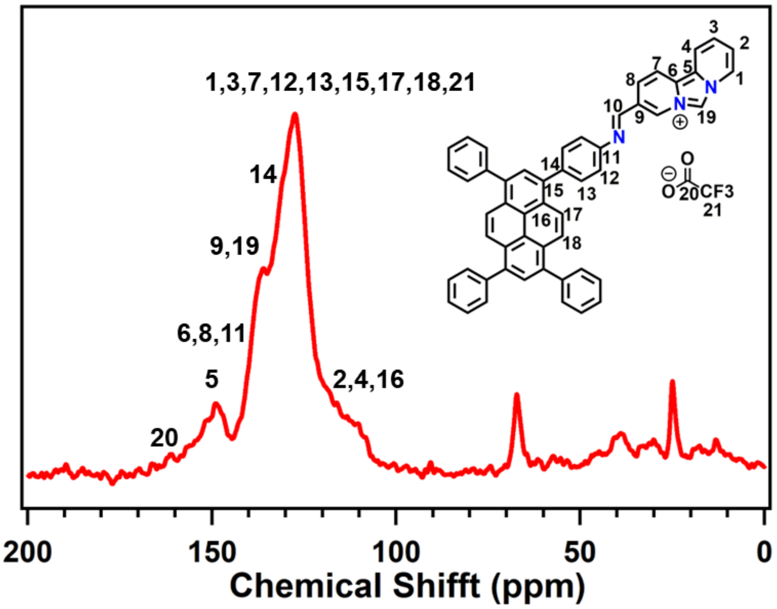

Supplementary Fig. 10 The ^{13}C NMR spectra of *im*-PY-BPY-COF.

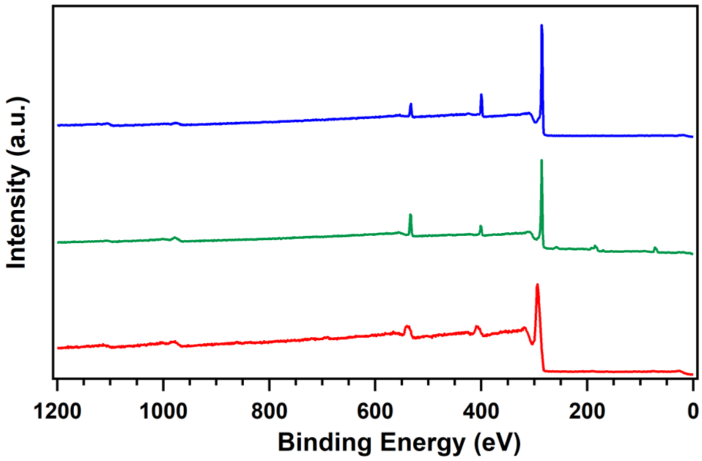

Supplementary Fig. 11 XPS spectra for PY-BPY-COF (blue), *ion*-PY-BPY-COF (green), *im*-PY-BPY-COF (red).

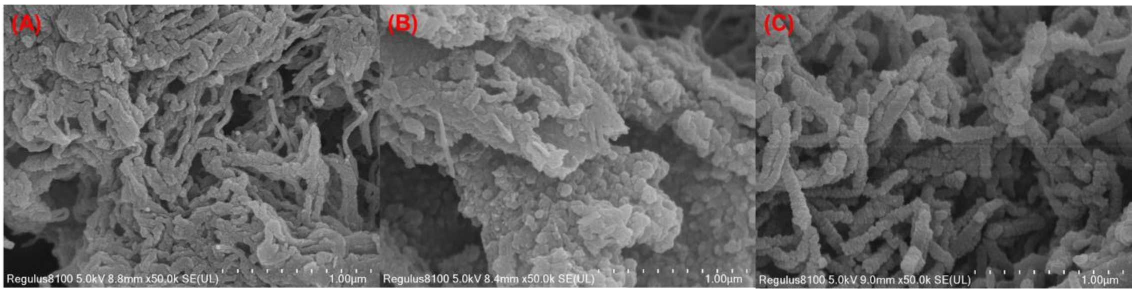

Supplementary Fig. 12 The FE-SEM images of (A) PY-BPY-COF, (B) *ion*-PY-BPY-COF, and (C) *im*-PY-BPY-COF.

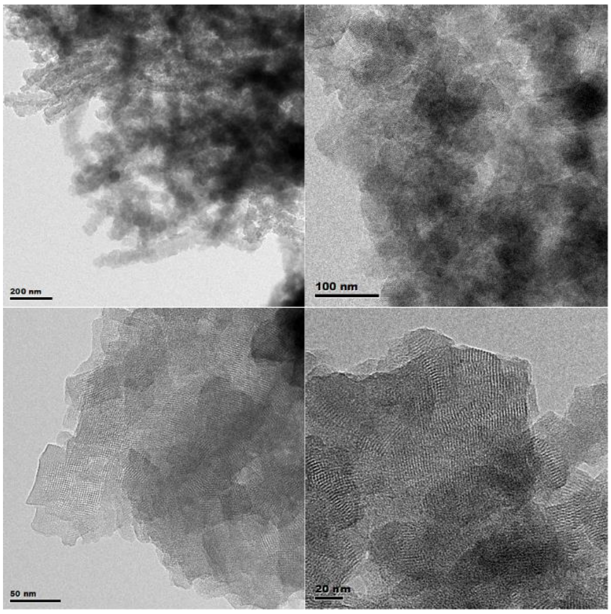

Supplementary Fig. 13 The TEM images of PY-BPY-COF with different scales.

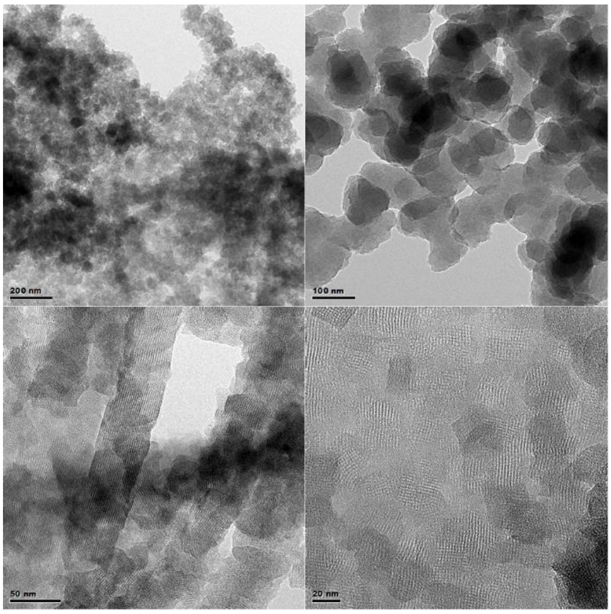

Supplementary Fig. 14 The TEM images of *ion*-PY-BPY-COF with different scales.

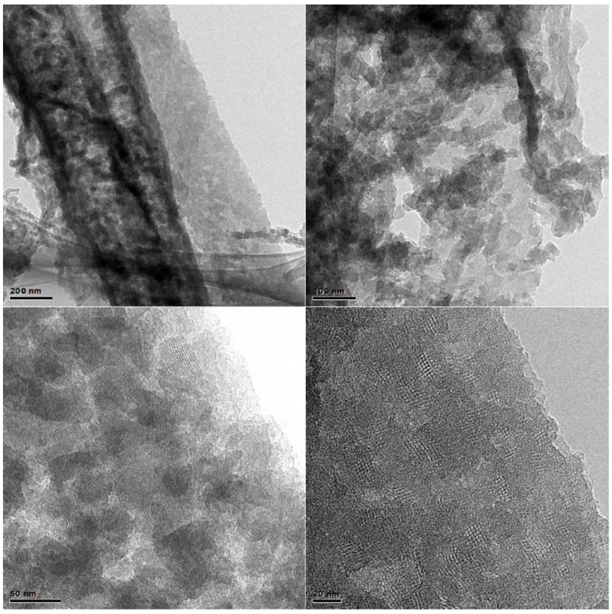

Supplementary Fig. 15 The TEM images of *im*-PY-BPY-COF with different scales.

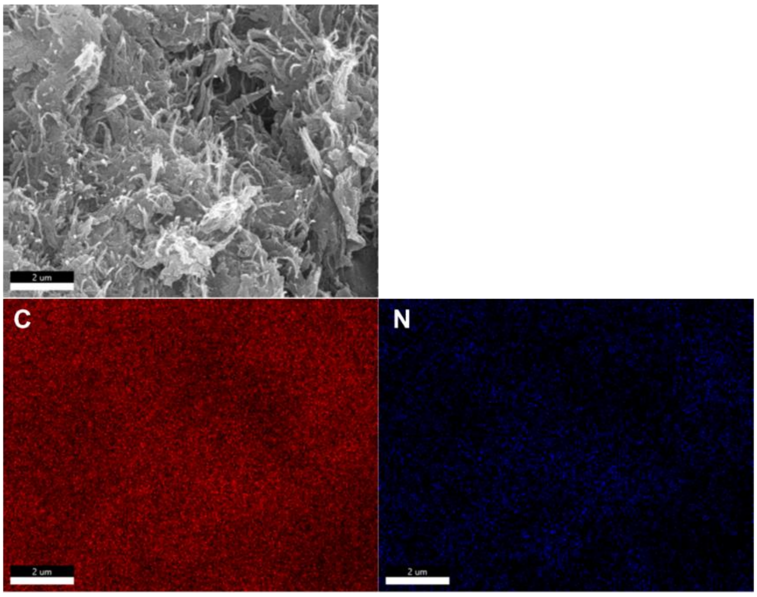

Supplementary Fig. 16 The EDX mapping images of PY-BPY-COF.

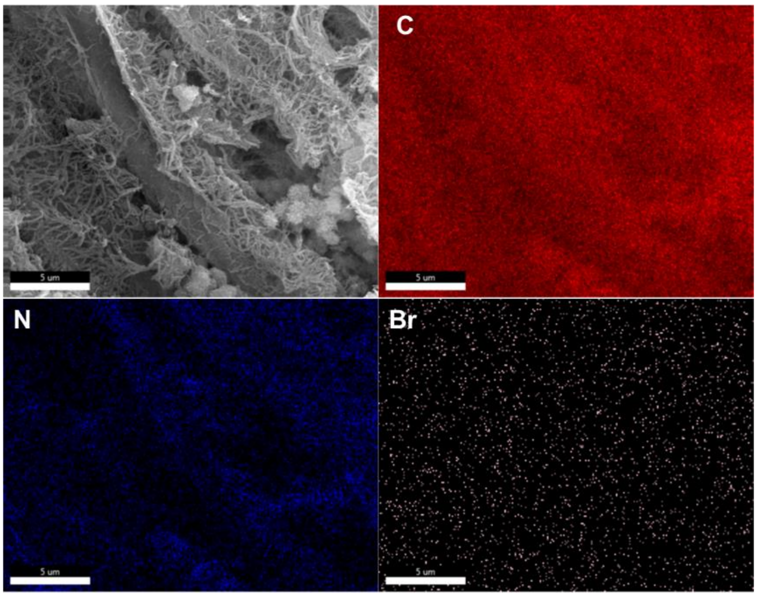

Supplementary Fig. 17 The EDX mapping images of *ion*-PY-BPY-COF.

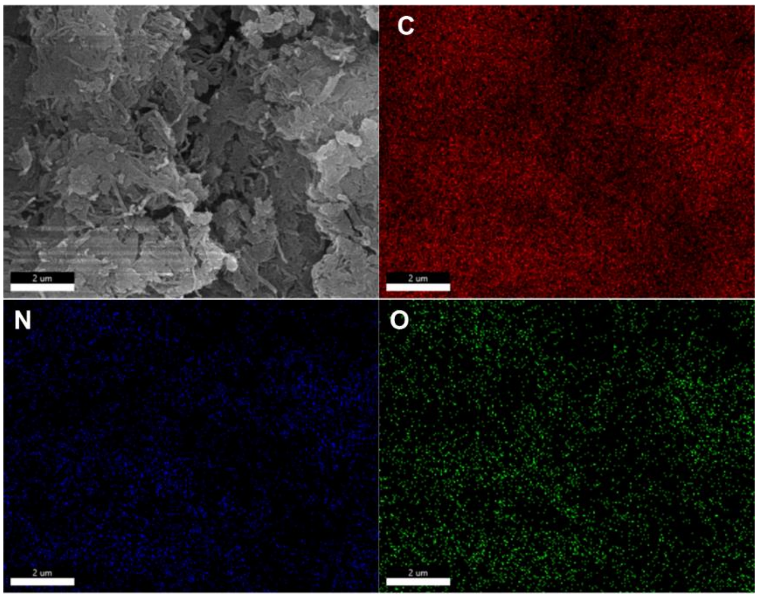

Supplementary Fig. 18 The EDX mapping images of *im*-PY-BPY-COF.

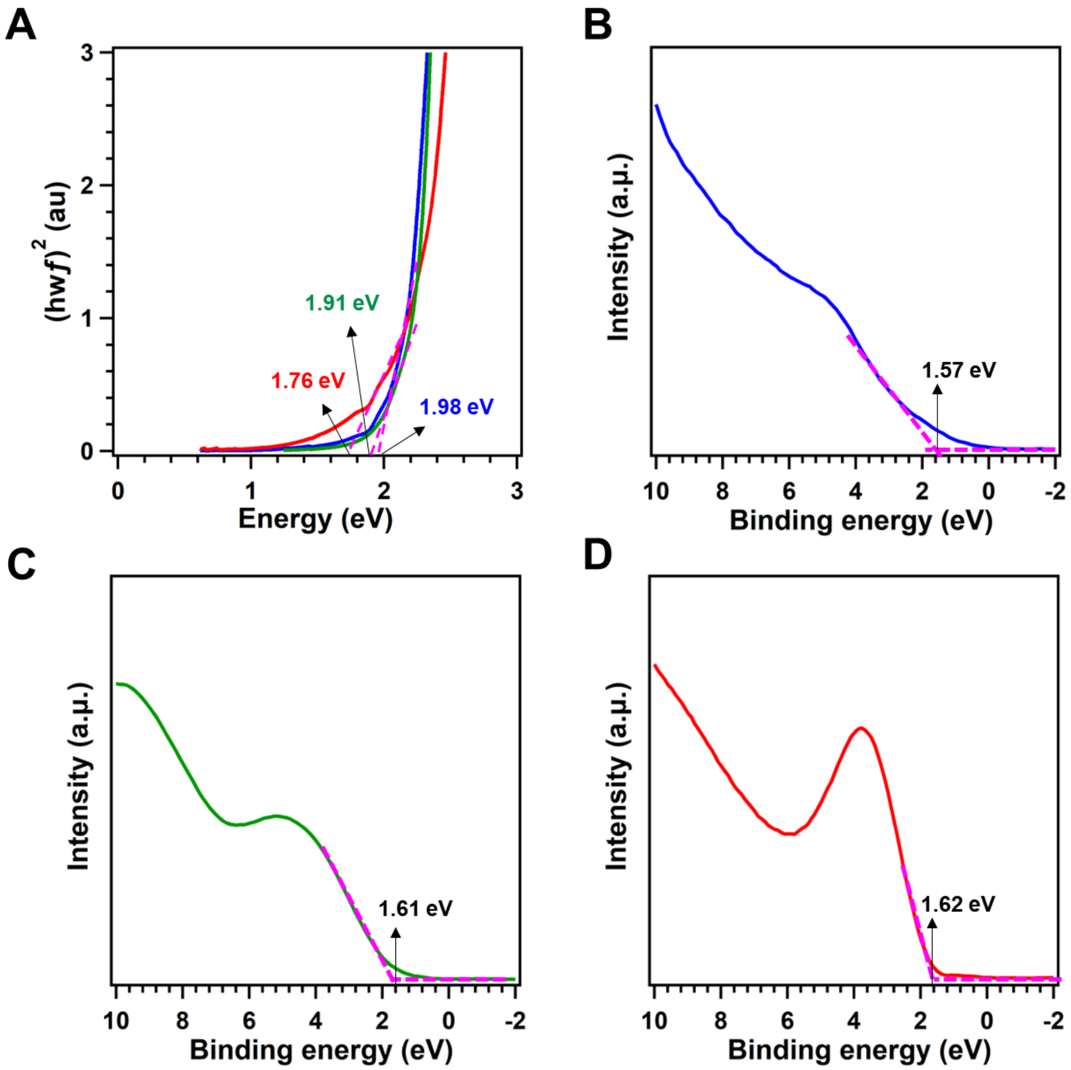

Supplementary Fig. 19 (A) The tauc plots; valence band positions of the (B) PY-BPY-COF, (C) *ion*-PY-BPY-COF, and (D) *im*-PY-BPY-COF.

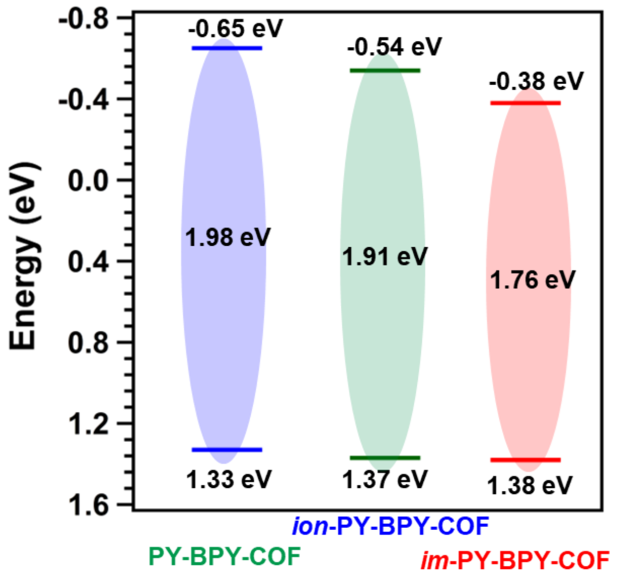

Supplementary Fig. 20 The band positions of the PY-BPY-COF, *ion*-PY-BPY-COF, and *im*-PY-BPY-COF.

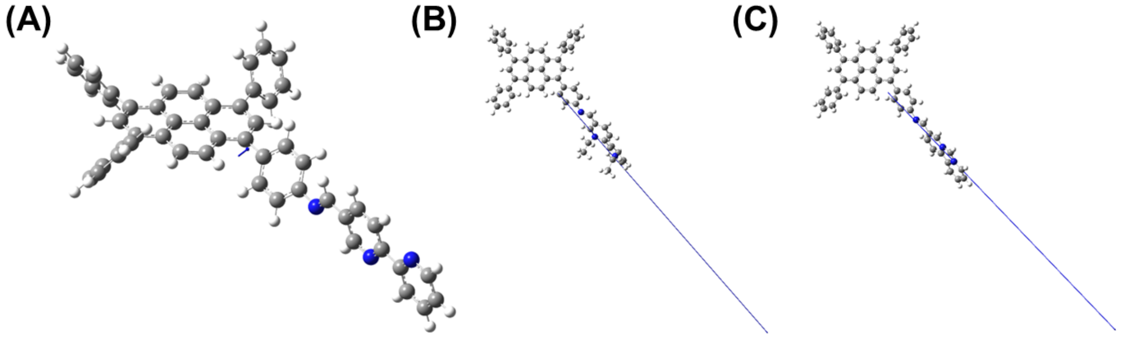

Supplementary Fig. 21 The dipole moments for (A) PY-BPY-COF, (B) *ion*-PY-BPY-COF, and (C) *im*-PY-BPY-COF.

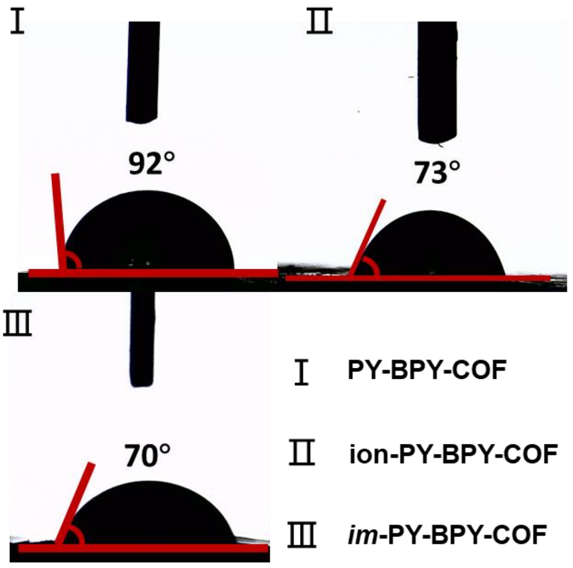

Supplementary Fig. 22 water contact angle photo for the three COFs.

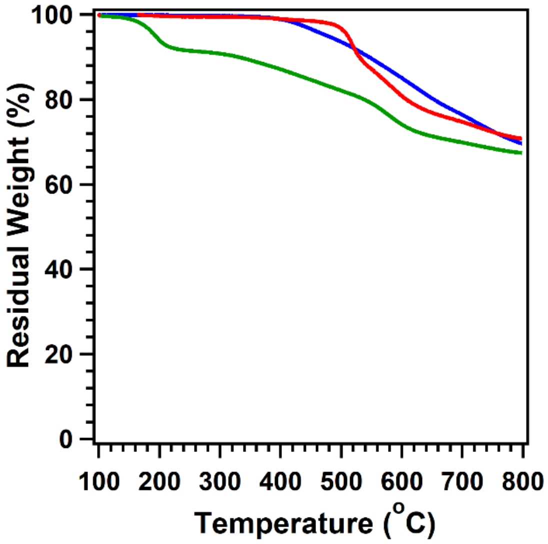

Supplementary Fig. 23 The TGA curves for PY-BPY-COF (blue), *ion*-PY-BPY-COF (green), and *im*-PY-BPY-COF (red) from 100 to 800 °C under N_2 atmosphere.

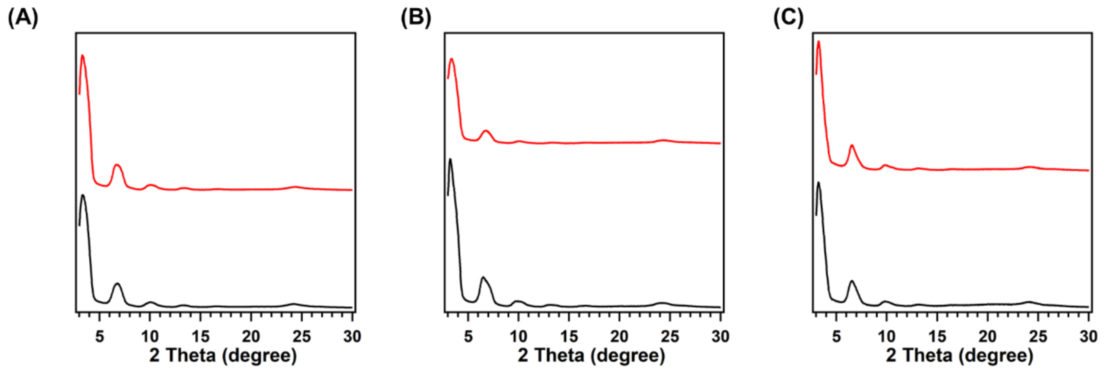

Supplementary Fig. 24 The PXRD patterns for (A) PY-BPY-COF, (B) *ion*-PY-BPY-COF, and (C) *im*-PY-BPY-COF before (black curves) and after (red curves) treated with the 0.1 M KOH aqueous solutions.

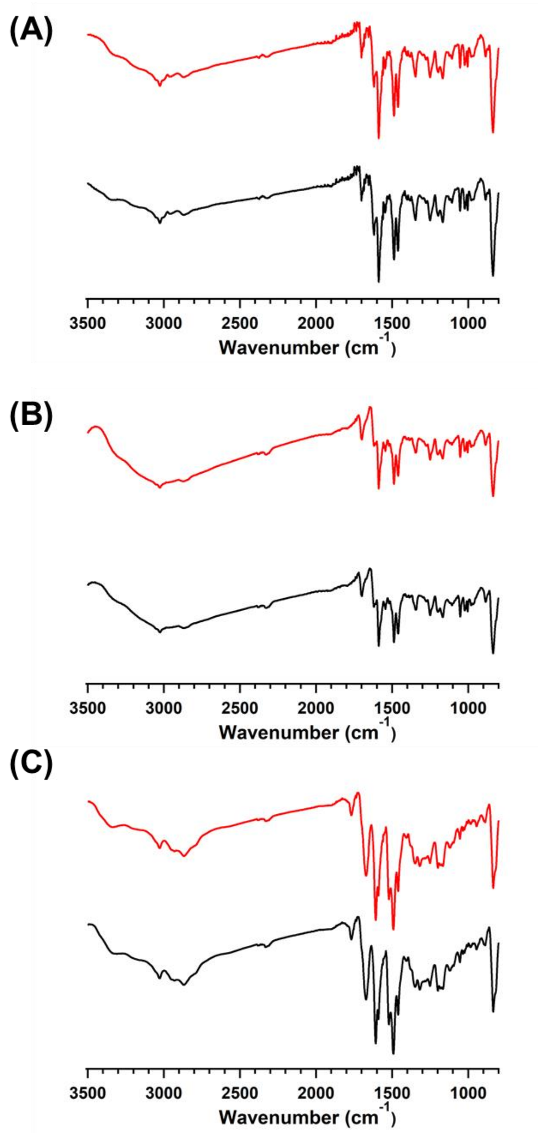

Supplementary Fig. 25 The FT-IR spectrum for (A) PY-BPY-COF, (B) *ion*-PY-BPY-COF, and (C) *im*-PY-BPY-COF before (black curves) and after (red curves) soaking the 0.1 M KOH aqueous solutions.

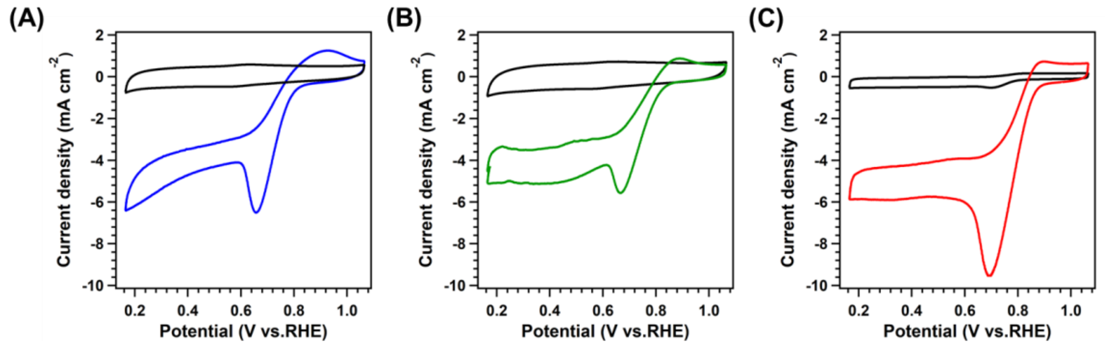

Supplementary Fig. 26 The CV curves for (A) PY-BPY-COF, (B) *ion*-PY-BPY-COF, and (C) *im*-PY-BPY-COF in O_2-staturated and N_2-staturaed KOH (0.1 M) aqueous solutions.

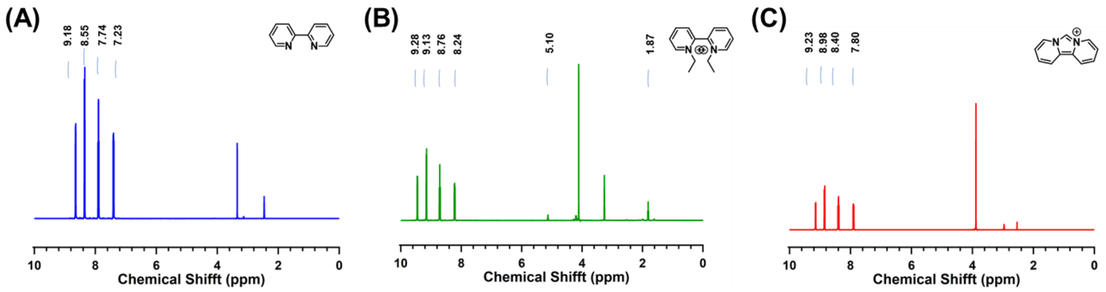

Supplementary Fig. 27 ^{1}H NMR spectrum of (A) BPY (blue), (B) *ion*-BPY (greed), and (C) *im*-BPY (red) recorded in DMSO-d_6 (T=298K, ppm).

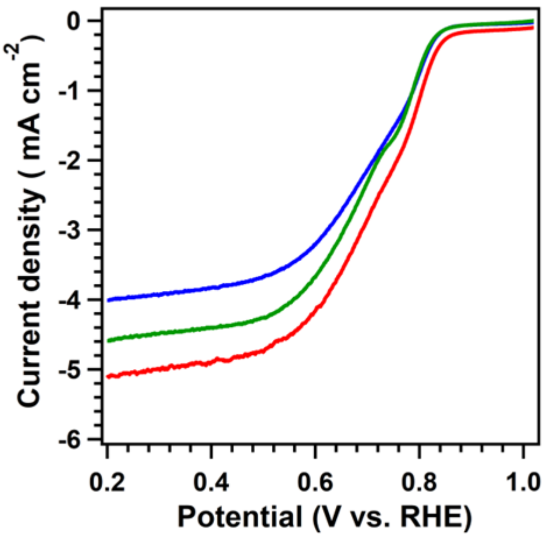

Supplementary Fig. 28 The LSV curves of BPY (blue), *ion*-BPY (greed), and *im*-BPY (red).

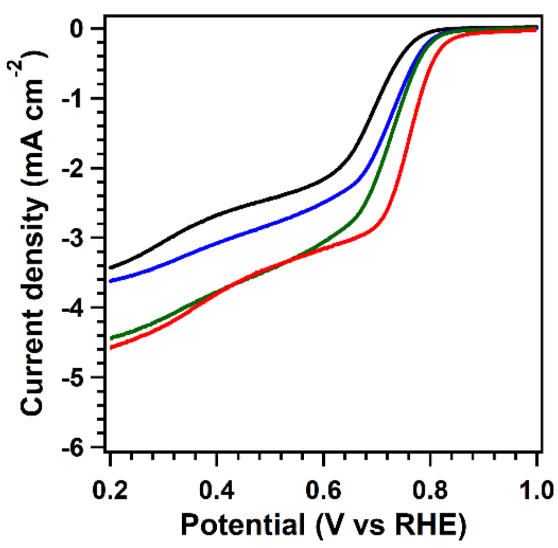

Supplementary Fig. 29 The LSV curves of amination CNT (black), BPY-CNT (blue), *ion*-BPY-CNT (green), and *im*-BPY-CNT (red).

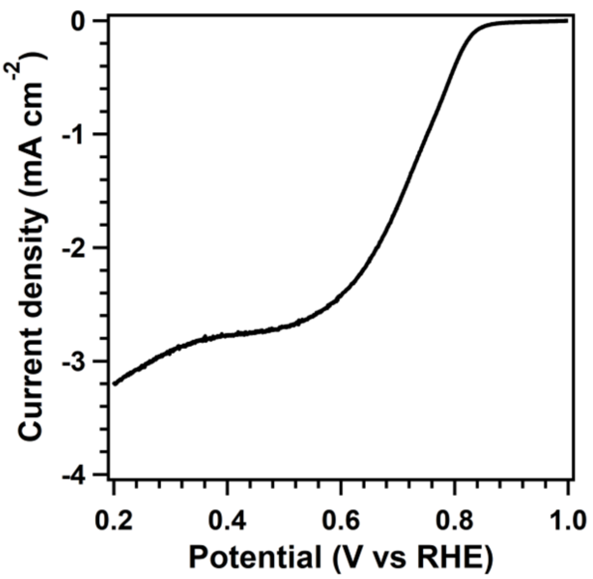

Supplementary Fig. 30 The LSV curve for the CNT in 0.1 M KOH.

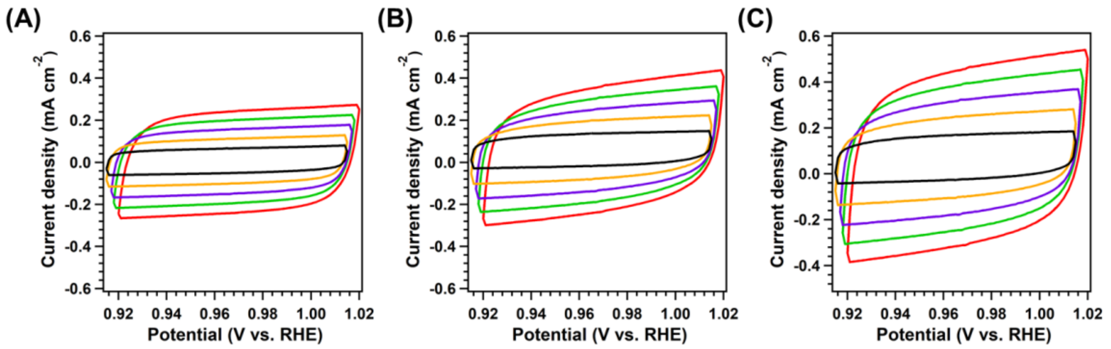

Supplementary Fig. 31 The CV measurements for (A) PY-BPY-COF, (B) *ion*-PY-BPY-COF, and (C) *im*-PY-BPY-COF at scan rates from 10 to 50 mV s^{-1}.

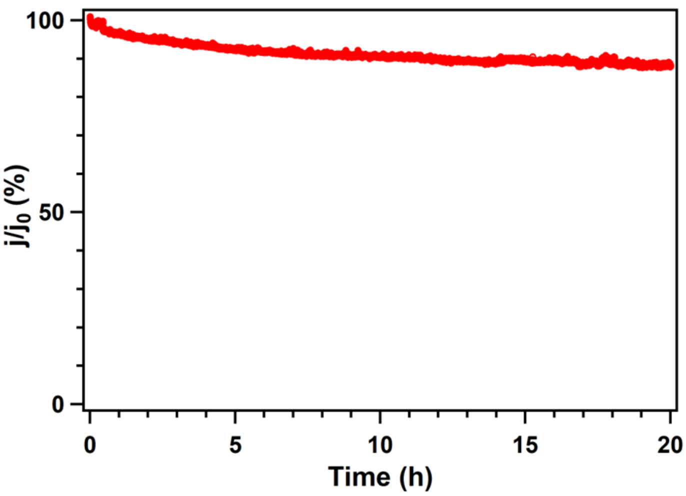

Supplementary Fig. 32 The long-term stability of *im*-PY-BPY-COF at 0.4 V in 0.1 M KOH solution for 20 h.

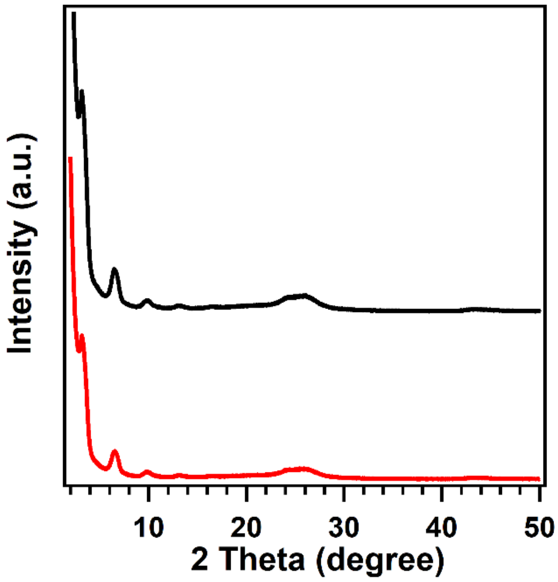

Supplementary Fig. 33 The XRD patterns of *im*-PY-BPY-COF before (black) and after (red) the chronoamperometry test.

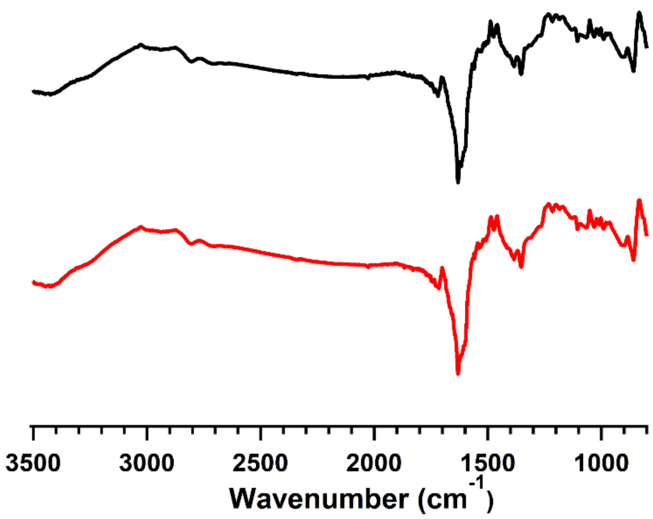

Supplementary Fig. 34 The FT-IR spectra of *im*-PY-BPY-COF before (black) and after (red) the chronoamperometry test.

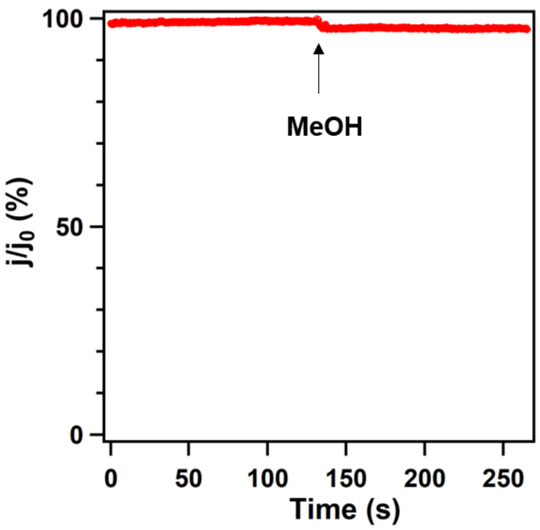

Supplementary Fig. 35 The long-term stability of *im*-PY-BPY-COF with addition of MeOH in 0.1 M KOH solutions.

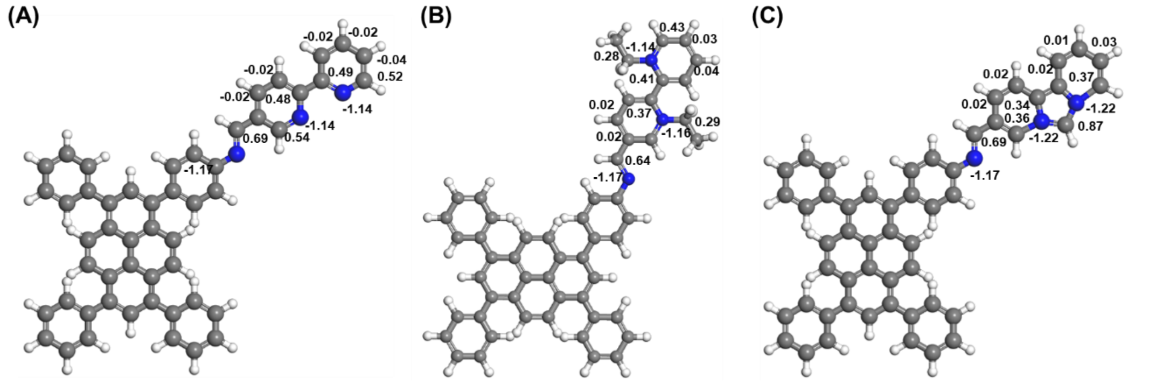

Supplementary Fig. 36 The Bader chargers of (A) PY-BPY-COF, (B) *ion*-PY-BPY-COF, and (C) *im*-PY-BPY-COF.

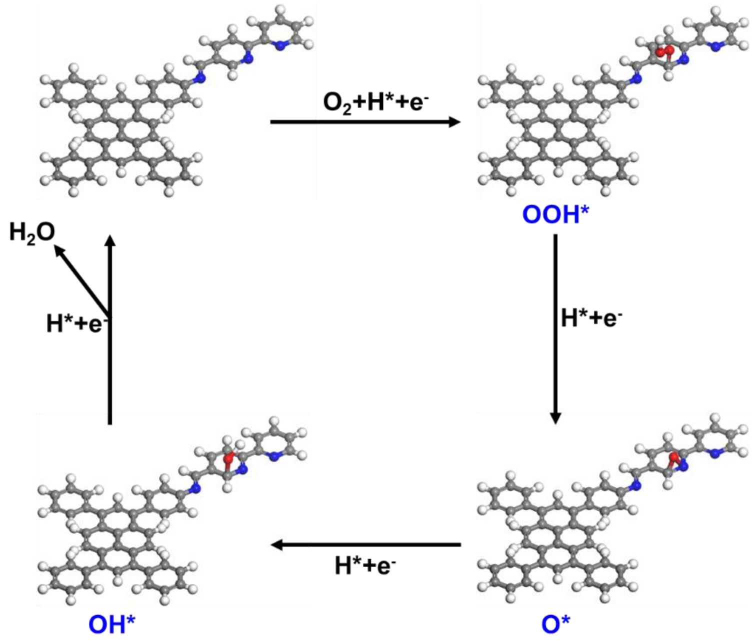

Supplementary Fig. 37 The ORR process structure diagram of PY-BPY-COF.

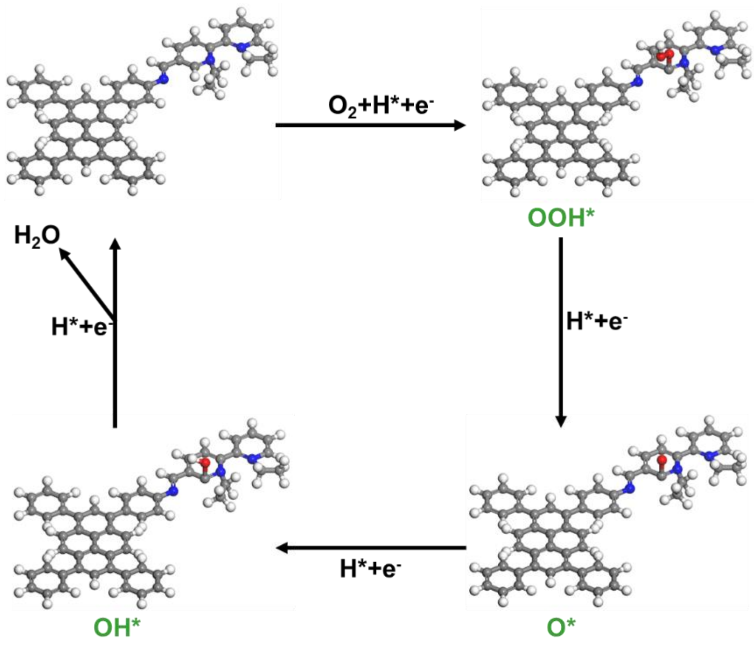

Supplementary Fig. 38 The ORR process structure diagram of *ion*-PY-BPY-COF.

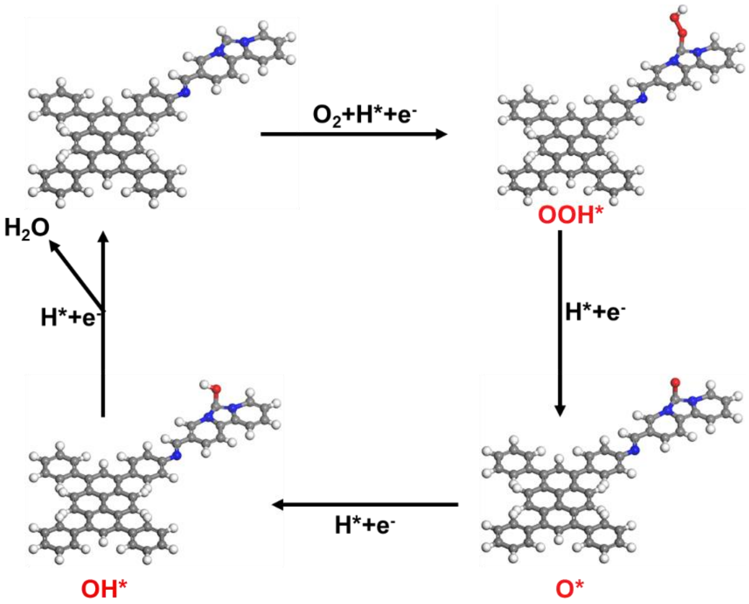

Supplementary Fig. 39 The ORR process structure diagram of *im*-PY-BPY-COF.

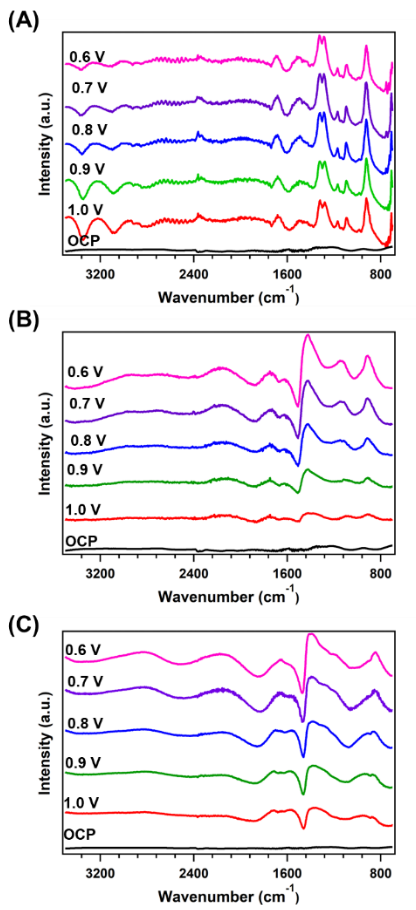

Supplementary Fig. 40 *In-situ* SR-FTIR spectroscopy measurement for (A) PY-BPY-COF, (B) *ion*-PY-BPY-COF, and (C) *im*-PY-BPY-COF electrocatalyst during the ORR process under various potentials.

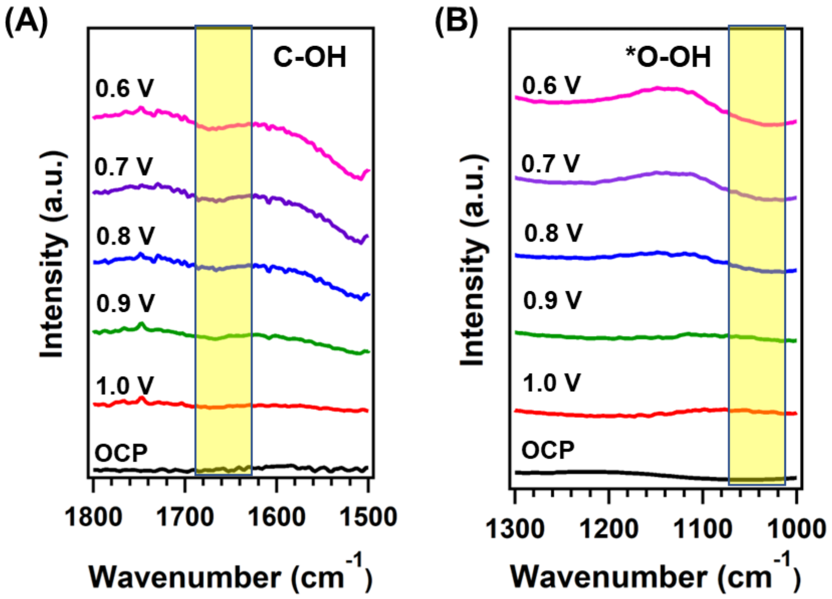

Supplementary Fig. 41 (A) *In-situ* SR-FTIR spectroscopy results in the range of 1800-1500 cm^{-1} and (B) 1300-1000 cm^{-1} at typical potentials of 0.6 V to 1.0 V for *ion*-PY-BPY-COF.

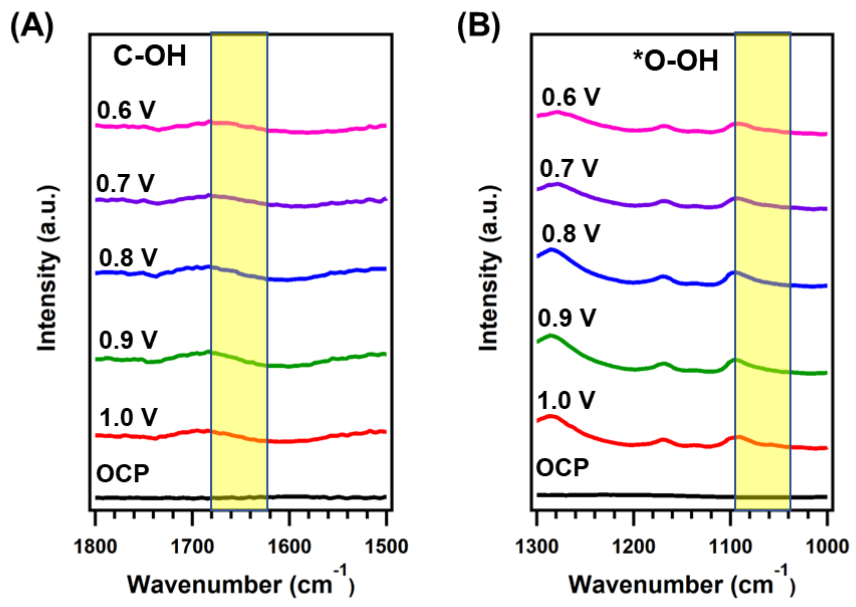

Supplementary Fig. 42 (A) *In-situ* SR-FTIR spectroscopy results in the range of 1800-1500 cm^{-1} and (B) 1300-1000 cm^{-1} at typical potentials of 0.6 V to 1.0 V for PY-BPY-COF.

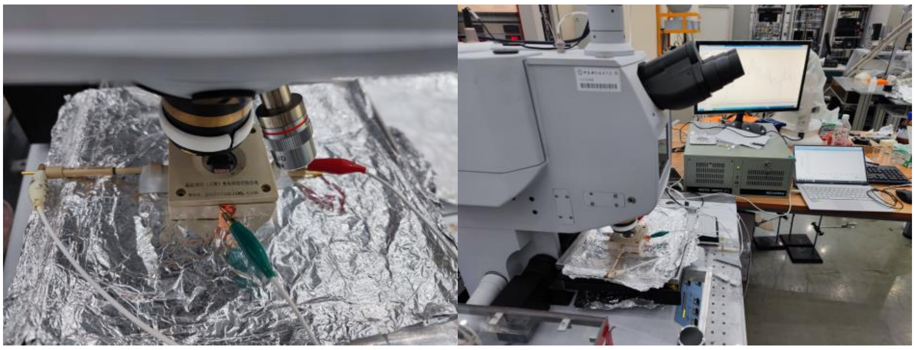

Supplementary Fig. 43 The diagram of electrochemical cells.

Supporting Tables:

Supplementary Table 1. Atomistic coordinates for the AA-stacking mode of *ion*-PY-BPY-COF optimized using DFTB+ method. Lattice type: orthorhombic, Space group: PMMM; α= β= γ = 90°, a = 39.715061 Å, b = 39.434367 Å, c = 3.766211 Å.

C1	C	0.40749	1.00324	1.02457	0	Uiso	1
C2	C	0.43901	1.02318	1.10595	0	Uiso	1
C3	C	0.4685	1.0057	1.21343	0	Uiso	1
C4	C	0.4995	1.02335	1.24994	0	Uiso	1
C5	C	0.53159	1.00445	1.3449	0	Uiso	1
C6	C	0.44026	1.05864	1.04742	0	Uiso	1
C7	C	0.47115	1.07524	1.0858	0	Uiso	1
C8	C	0.50075	1.05853	1.17984	0	Uiso	1
C9	C	0.4107	1.07968	0.93966	0	Uiso	1
C10	C	0.53234	1.07882	1.18227	0	Uiso	1
C11	C	0.37738	1.06884	0.99209	0	Uiso	1
C12	C	0.3502	1.08941	0.896	0	Uiso	1
C13	C	0.3558	1.12141	0.74827	0	Uiso	1
C14	C	0.38897	1.13263	0.6976	0	Uiso	1
C15	C	0.41602	1.112	0.79283	0	Uiso	1
C16	C	0.53132	1.11331	1.27536	0	Uiso	1
C17	C	0.56026	1.13336	1.25493	0	Uiso	1
C18	C	0.59083	1.11927	1.13894	0	Uiso	1
C19	C	0.59195	1.08501	1.04503	0	Uiso	1
C20	C	0.56298	1.06503	1.06518	0	Uiso	1
N21	N	0.32754	1.142	0.6519	0	Uiso	1
N22	N	0.62058	1.13943	1.09858	0	Uiso	1
C23	C	0.79843	1.25191	0.71922	0	Uiso	1
C24	C	0.76957	1.23512	0.82596	0	Uiso	1
C25	C	0.73946	1.25273	0.8971	0	Uiso	1
N26	N	0.73752	1.28743	0.86697	0	Uiso	1
C27	C	0.76655	1.30435	0.7604	0	Uiso	1
C28	C	0.79757	1.2867	0.68197	0	Uiso	1
C29	C	0.7088	1.23223	0.98658	0	Uiso	1
C30	C	0.70941	1.19738	0.90658	0	Uiso	1

C31	C	0.68145	1.1771	0.97199	0	Uiso	1
C32	C	0.65253	1.19139	1.11864	0	Uiso	1
C33	C	0.65259	1.2258	1.20198	0	Uiso	1
N34	N	0.68005	1.2457	1.13646	0	Uiso	1
C35	C	0.82759	1.30485	0.55865	0	Uiso	1
C36	C	0.26894	1.18176	0.4769	0	Uiso	1
C37	C	0.2422	1.20273	0.38469	0	Uiso	1
C38	C	0.24812	1.23436	0.225	0	Uiso	1
N39	N	0.28072	1.24388	0.14853	0	Uiso	1
C40	C	0.30702	1.22336	0.24343	0	Uiso	1
C41	C	0.30177	1.19229	0.40942	0	Uiso	1
C42	C	0.21786	1.25488	0.12591	0	Uiso	1
C43	C	0.19132	1.23779	-0.04376	0	Uiso	1
C44	C	0.1613	1.25454	-0.11802	0	Uiso	1
C45	C	0.15747	1.28829	-0.01998	0	Uiso	1
C46	C	0.18392	1.30462	0.15368	0	Uiso	1
N47	N	0.21344	1.28828	0.22364	0	Uiso	1
C48	C	0.00187	1.45155	-0.12793	0	Uiso	1
C49	C	0.00019	1.41394	-0.22676	0	Uiso	1
C50	C	0.00022	1.52349	-0.24147	0	Uiso	1
C51	C	0.02959	1.50331	-0.35674	0	Uiso	1
C52	C	0.03002	1.46971	-0.31768	0	Uiso	1
C53	C	0.00189	1.55909	-0.20412	0	Uiso	1
C54	C	0.03378	1.5789	-0.21868	0	Uiso	1
C55	C	0.03213	1.39404	-0.16081	0	Uiso	1
C56	C	0.03284	1.61366	-0.30019	0	Uiso	1
C57	C	0.06179	1.63356	-0.27475	0	Uiso	1
C58	C	0.09239	1.61909	-0.16574	0	Uiso	1
C59	C	0.09361	1.58444	-0.08826	0	Uiso	1
C60	C	0.06456	1.56456	-0.11318	0	Uiso	1
C61	C	0.06029	1.40839	0.00718	0	Uiso	1
C62	C	0.08957	1.38947	0.05825	0	Uiso	1
C63	C	0.09113	1.35577	-0.05477	0	Uiso	1
C64	C	0.06298	1.34108	-0.21976	0	Uiso	1
C65	C	0.03378	1.36018	-0.27347	0	Uiso	1
N66	N	0.12151	1.33715	0.00369	0	Uiso	1

N67	N	0.12187	1.63936	-0.10961	0	Uiso	1
C68	C	0.3309	1.17151	0.50497	0	Uiso	1
C69	C	0.62186	1.17141	1.17658	0	Uiso	1
C70	C	0.1257	1.30586	-0.09136	0	Uiso	1
C71	C	0.94031	1.41554	0.00535	0	Uiso	1
C72	C	0.93967	1.45125	-0.05953	0	Uiso	1
C73	C	0.96816	1.46797	-0.22142	0	Uiso	1
C74	C	0.97108	1.39669	-0.03106	0	Uiso	1
C75	C	0.91065	1.47022	0.01163	0	Uiso	1
C76	C	0.90973	1.50528	-0.01009	0	Uiso	1
C77	C	0.9403	1.52469	-0.09203	0	Uiso	1
C78	C	0.96955	1.50638	-0.17792	0	Uiso	1
C79	C	0.94139	1.56049	-0.06969	0	Uiso	1
C80	C	0.97233	1.57671	-0.12265	0	Uiso	1
C81	C	0.9118	1.58208	0.02064	0	Uiso	1
C82	C	0.91077	1.39556	0.1438	0	Uiso	1
C83	C	0.87862	1.571	-0.03497	0	Uiso	1
C84	C	0.85129	1.59186	0.04807	0	Uiso	1
C85	C	0.85665	1.62429	0.18637	0	Uiso	1
C86	C	0.88971	1.6358	0.23878	0	Uiso	1
C87	C	0.91693	1.61489	0.15606	0	Uiso	1
C88	C	0.91246	1.35989	0.15572	0	Uiso	1
C89	C	0.88547	1.34056	0.28077	0	Uiso	1
C90	C	0.85593	1.35649	0.39777	0	Uiso	1
C91	C	0.85396	1.3918	0.38952	0	Uiso	1
C92	C	0.88106	1.41101	0.26528	0	Uiso	1
N93	N	0.82821	1.64509	0.27086	0	Uiso	1
N94	N	0.82756	1.33755	0.52329	0	Uiso	1
C95	C	0.76935	1.68565	0.41023	0	Uiso	1
C96	C	0.74231	1.70662	0.49266	0	Uiso	1
C97	C	0.74769	1.73794	0.66042	0	Uiso	1
N98	N	0.78006	1.74718	0.7503	0	Uiso	1
C99	C	0.80659	1.72645	0.67252	0	Uiso	1
C100	C	0.8019	1.6957	0.49895	0	Uiso	1
C101	C	0.71715	1.75777	0.76278	0	Uiso	1
C102	C	0.69076	1.73985	0.92537	0	Uiso	1

C103	C	0.66049	1.75593	1.00344	0	Uiso	1
C104	C	0.6563	1.78989	0.91637	0	Uiso	1
C105	C	0.68259	1.80705	0.74831	0	Uiso	1
N106	N	0.71234	1.79132	0.67437	0	Uiso	1
C107	C	0.29679	1.75463	0.34307	0	Uiso	1
C108	C	0.26819	1.73767	0.23158	0	Uiso	1
C109	C	0.23719	1.75458	0.19218	0	Uiso	1
N110	N	0.23404	1.78866	0.26103	0	Uiso	1
C111	C	0.2628	1.80578	0.37208	0	Uiso	1
C112	C	0.29477	1.78882	0.4173	0	Uiso	1
C113	C	0.20706	1.73383	0.09057	0	Uiso	1
C114	C	0.20871	1.69858	0.14418	0	Uiso	1
C115	C	0.18143	1.67794	0.06004	0	Uiso	1
C116	C	0.1523	1.6923	-0.08079	0	Uiso	1
C117	C	0.15135	1.72716	-0.13757	0	Uiso	1
N118	N	0.17805	1.74743	-0.05183	0	Uiso	1
C119	C	0.32471	1.80698	0.54183	0	Uiso	1
C120	C	0.1223	1.67192	-0.15791	0	Uiso	1
C121	C	0.83126	1.6747	0.41678	0	Uiso	1
C122	C	0.43838	1.91614	1.08413	0	Uiso	1
C123	C	0.43768	1.95089	1.17954	0	Uiso	1
C124	C	0.50056	1.95188	1.16025	0	Uiso	1
C125	C	0.50001	1.91558	1.14874	0	Uiso	1
C126	C	0.46937	1.89923	1.08795	0	Uiso	1
C127	C	0.40802	1.89624	0.97371	0	Uiso	1
C128	C	0.53137	1.89554	1.10035	0	Uiso	1
C129	C	0.56096	1.91111	0.97845	0	Uiso	1
C130	C	0.5902	1.89225	0.92487	0	Uiso	1
C131	C	0.59043	1.85747	0.99176	0	Uiso	1
C132	C	0.56097	1.84155	1.11304	0	Uiso	1
C133	C	0.53165	1.86049	1.16608	0	Uiso	1
C134	C	0.41028	1.86082	0.93601	0	Uiso	1
C135	C	0.38282	1.8418	0.81839	0	Uiso	1
C136	C	0.35233	1.85785	0.73452	0	Uiso	1
C137	C	0.34977	1.893	0.77097	0	Uiso	1
C138	C	0.37738	1.91197	0.88742	0	Uiso	1

N139	N	0.32378	1.83918	0.60977	0	Uiso	1
N140	N	0.62079	1.83899	0.92834	0	Uiso	1
C141	C	0.62442	1.80696	0.99432	0	Uiso	1
C142	C	0.46775	1.96788	1.29165	0	Uiso	1
C143	C	0.40562	1.97012	1.2294	0	Uiso	1
C144	C	0.52791	1.96619	1.39976	0	Uiso	1
C145	C	0.17485	1.78334	-0.1383	0	Uiso	1
C146	C	0.14535	1.79926	0.05798	0	Uiso	1
C147	C	0.24009	1.82463	0.35287	0	Uiso	1
C148	C	0.20718	1.84565	0.3232	0	Uiso	1
C149	C	0.73683	1.81049	0.46708	0	Uiso	1
C150	C	0.74633	1.84354	0.65005	0	Uiso	1
C151	C	0.78733	1.77733	0.96103	0	Uiso	1
C152	C	0.8135	1.79976	0.78177	0	Uiso	1
C153	C	0.74497	1.32383	0.81104	0	Uiso	1
C154	C	0.71338	1.34594	0.88118	0	Uiso	1
C155	C	0.6778	1.28094	1.25181	0	Uiso	1
C156	C	0.64962	1.29943	1.05829	0	Uiso	1
C157	C	0.2875	1.27514	-0.05059	0	Uiso	1
C158	C	0.32476	1.28227	-0.11795	0	Uiso	1
C159	C	0.23801	1.30667	0.43734	0	Uiso	1
C160	C	0.24969	1.3389	0.25313	0	Uiso	1

Supplementary Table 2 Atomistic coordinates for the AA-stacking mode of *im*-PY-BPY-COF optimized using DFTB+ method. Lattice type: orthorhombic, Space group: PMMM; α= β= γ = 90°, a = 38.558292 Å, b = 39.310914 Å, c = 4.046332 Å.

C1	C	0.42106	1.03049	0.49585	0	Uiso	1
C2	C	0.45468	1.05005	0.47564	0	Uiso	1
C3	C	0.4848	1.03391	0.35135	0	Uiso	1
C4	C	0.51709	1.05106	0.35561	0	Uiso	1
C5	C	0.55001	1.03325	0.23993	0	Uiso	1
C6	C	0.45653	1.08419	0.58602	0	Uiso	1
C7	C	0.48864	1.10076	0.58446	0	Uiso	1
C8	C	0.51893	1.08458	0.47998	0	Uiso	1
C9	C	0.42554	1.10381	0.69309	0	Uiso	1
C10	C	0.55225	1.10294	0.51527	0	Uiso	1
C11	C	0.39877	1.08853	0.87476	0	Uiso	1
C12	C	0.36876	1.10663	0.95507	0	Uiso	1
C13	C	0.36501	1.14045	0.85592	0	Uiso	1
C14	C	0.39205	1.15627	0.68177	0	Uiso	1
C15	C	0.42213	1.1381	0.60253	0	Uiso	1
C16	C	0.55555	1.13679	0.41034	0	Uiso	1
C17	C	0.58728	1.15379	0.44227	0	Uiso	1
C18	C	0.61599	1.1371	0.58212	0	Uiso	1
C19	C	0.61236	1.10368	0.69333	0	Uiso	1
C20	C	0.58079	1.0868	0.66106	0	Uiso	1
N21	N	0.33278	1.15738	0.91759	0	Uiso	1
N22	N	0.64757	1.1519	0.60555	0	Uiso	1
C23	C	0.8455	1.25488	0.55134	0	Uiso	1
C24	C	0.81956	1.23079	0.61811	0	Uiso	1
C25	C	0.78591	1.23904	0.52325	0	Uiso	1
N26	N	0.77848	1.26876	0.37302	0	Uiso	1
C27	C	0.80282	1.29265	0.30371	0	Uiso	1
C28	C	0.83738	1.28586	0.39434	0	Uiso	1
C29	C	0.75571	1.22085	0.55068	0	Uiso	1
C30	C	0.74973	1.18863	0.68019	0	Uiso	1
C31	C	0.71563	1.17638	0.66456	0	Uiso	1
C32	C	0.68882	1.19618	0.52316	0	Uiso	1
C33	C	0.69664	1.22862	0.39805	0	Uiso	1

N34	N	0.72996	1.23955	0.41739	0	Uiso	1
C35	C	0.86533	1.31021	0.33066	0	Uiso	1
C36	C	0.26467	1.18537	1.02352	0	Uiso	1
C37	C	0.23125	1.19939	1.04595	0	Uiso	1
C38	C	0.22654	1.2317	0.91273	0	Uiso	1
N39	N	0.25288	1.24878	0.77053	0	Uiso	1
C40	C	0.28545	1.23608	0.74301	0	Uiso	1
C41	C	0.292	1.20359	0.8718	0	Uiso	1
C42	C	0.19708	1.25105	0.88098	0	Uiso	1
C43	C	0.16298	1.24429	0.97274	0	Uiso	1
C44	C	0.13795	1.26878	0.88875	0	Uiso	1
C45	C	0.14733	1.29867	0.71841	0	Uiso	1
C46	C	0.18231	1.30395	0.63359	0	Uiso	1
N47	N	0.20572	1.27979	0.71953	0	Uiso	1
C48	C	0.02346	1.47553	0.22777	0	Uiso	1
C49	C	0.03038	1.44572	–0.00767	0	Uiso	1
C50	C	0.01616	1.54688	0.38775	0	Uiso	1
C51	C	0.05065	1.5318	0.32019	0	Uiso	1
C52	C	0.05423	1.49905	0.23971	0	Uiso	1
C53	C	0.01255	1.57975	0.52351	0	Uiso	1
C54	C	0.04271	1.60135	0.60756	0	Uiso	1
C55	C	0.05598	1.42044	0.12553	0	Uiso	1
C56	C	0.04288	1.63608	0.52655	0	Uiso	1
C57	C	0.07191	1.65617	0.59751	0	Uiso	1
C58	C	0.10091	1.6417	0.75413	0	Uiso	1
C59	C	0.10034	1.60736	0.84095	0	Uiso	1
C60	C	0.0715	1.58737	0.76833	0	Uiso	1
C61	C	0.09137	1.42547	0.06776	0	Uiso	1
C62	C	0.11566	1.40194	0.18325	0	Uiso	1
C63	C	0.1049	1.37297	0.35581	0	Uiso	1
C64	C	0.06935	1.36784	0.41285	0	Uiso	1
C65	C	0.04508	1.39144	0.29843	0	Uiso	1
N66	N	0.12913	1.35041	0.4591	0	Uiso	1
N67	N	0.13018	1.65961	0.81485	0	Uiso	1
C68	C	0.32625	1.1882	0.82167	0	Uiso	1
C69	C	0.65528	1.1836	0.49768	0	Uiso	1
C70	C	0.12165	1.32167	0.62874	0	Uiso	1

C71	C	0.96551	1.43908	–0.00789	0	Uiso	1
C72	C	0.96129	1.4727	0.10987	0	Uiso	1
C73	C	0.99094	1.49485	0.11619	0	Uiso	1
C74	C	0.99766	1.4286	–0.15131	0	Uiso	1
C75	C	0.92883	1.48422	0.21625	0	Uiso	1
C76	C	0.9239	1.51667	0.34098	0	Uiso	1
C77	C	0.95322	1.54016	0.38643	0	Uiso	1
C78	C	0.98645	1.52781	0.30872	0	Uiso	1
C79	C	0.94918	1.57369	0.51267	0	Uiso	1
C80	C	0.97923	1.59271	0.57702	0	Uiso	1
C81	C	0.9148	1.59081	0.56067	0	Uiso	1
C82	C	0.93824	1.41286	0.02862	0	Uiso	1
C83	C	0.88352	1.57407	0.49165	0	Uiso	1
C84	C	0.85188	1.59106	0.50743	0	Uiso	1
C85	C	0.85062	1.6254	0.59227	0	Uiso	1
C86	C	0.88152	1.64251	0.67041	0	Uiso	1
C87	C	0.91326	1.62528	0.65456	0	Uiso	1
C88	C	0.94677	1.38129	0.16704	0	Uiso	1
C89	C	0.92129	1.35655	0.2137	0	Uiso	1
C90	C	0.88683	1.36299	0.12008	0	Uiso	1
C91	C	0.87846	1.3942	–0.02509	0	Uiso	1
C92	C	0.90397	1.41902	–0.07075	0	Uiso	1
N93	N	0.81968	1.64148	0.57507	0	Uiso	1
N94	N	0.85985	1.3387	0.17891	0	Uiso	1
C95	C	0.75483	1.66936	0.44184	0	Uiso	1
C96	C	0.72292	1.684	0.359	0	Uiso	1
C97	C	0.71925	1.71861	0.41843	0	Uiso	1
N98	N	0.74487	1.73722	0.55331	0	Uiso	1
C99	C	0.77629	1.72405	0.6349	0	Uiso	1
C100	C	0.7819	1.68917	0.57846	0	Uiso	1
C101	C	0.69208	1.73984	0.35259	0	Uiso	1
C102	C	0.66072	1.73262	0.20363	0	Uiso	1
C103	C	0.63822	1.75976	0.15938	0	Uiso	1
C104	C	0.64709	1.79276	0.2621	0	Uiso	1
C105	C	0.67994	1.80023	0.41841	0	Uiso	1
N106	N	0.70072	1.77152	0.44902	0	Uiso	1
C107	C	0.31882	1.7753	0.93646	0	Uiso	1

C108	C	0.2937	1.75028	0.99192	0	Uiso	1
C109	C	0.26032	1.75726	0.88134	0	Uiso	1
N110	N	0.25233	1.78671	0.72872	0	Uiso	1
C111	C	0.27596	1.81155	0.66985	0	Uiso	1
C112	C	0.31028	1.80599	0.77642	0	Uiso	1
C113	C	0.2313	1.7373	0.88497	0	Uiso	1
C114	C	0.22681	1.70429	0.99946	0	Uiso	1
C115	C	0.19414	1.68962	0.95096	0	Uiso	1
C116	C	0.16723	1.70784	0.79249	0	Uiso	1
C117	C	0.1735	1.74124	0.68374	0	Uiso	1
N118	N	0.20549	1.75459	0.7356	0	Uiso	1
C119	C	0.33837	1.82991	0.70913	0	Uiso	1
C120	C	0.13545	1.69247	0.72975	0	Uiso	1
C121	C	0.81425	1.67487	0.63736	0	Uiso	1
C122	C	0.45108	1.94481	0.28631	0	Uiso	1
C123	C	0.45141	1.98063	0.26553	0	Uiso	1
C124	C	0.51653	1.97897	0.29419	0	Uiso	1
C125	C	0.51458	1.94332	0.22438	0	Uiso	1
C126	C	0.4824	1.92747	0.24864	0	Uiso	1
C127	C	0.4198	1.92358	0.3556	0	Uiso	1
C128	C	0.54534	1.92055	0.22144	0	Uiso	1
C129	C	0.57714	1.93012	0.36352	0	Uiso	1
C130	C	0.60455	1.90677	0.38478	0	Uiso	1
C131	C	0.60044	1.8735	0.26662	0	Uiso	1
C132	C	0.56887	1.86427	0.11864	0	Uiso	1
C133	C	0.54179	1.88745	0.09662	0	Uiso	1
C134	C	0.42473	1.88992	0.46264	0	Uiso	1
C135	C	0.39675	1.86899	0.53463	0	Uiso	1
C136	C	0.3627	1.88108	0.50171	0	Uiso	1
C137	C	0.35729	1.91448	0.39229	0	Uiso	1
C138	C	0.38563	1.93559	0.32055	0	Uiso	1
N139	N	0.33373	1.85949	0.57442	0	Uiso	1
N140	N	0.62626	1.85043	0.30806	0	Uiso	1
C141	C	0.62232	1.81764	0.21323	0	Uiso	1
C142	C	0.48321	1.99844	0.20445	0	Uiso	1
C143	C	0.41895	2.00128	0.25137	0	Uiso	1
C144	C	0.54531	1.99698	0.10727	0	Uiso	1

C145	C	0.21856	1.78496	0.63933	0	Uiso	1
C146	C	0.24001	1.27833	0.65245	0	Uiso	1
C147	C	0.74406	1.26901	0.3085	0	Uiso	1
C148	C	0.73343	1.76969	0.5711	0	Uiso	1

Supplementary Table 3 Elemental analysis of PY-BPY-COF, *ion*-PY-BPY-COF, and *im*-PY-BPY-COF.

Samples		C	N	H
PY-BPY-COF	Cacld.	87.54%	7.56%	4.90%
	Found	85.11%	7.55%	4.62%
ion-PY-BPY-COF	Cacld.	79.25%	6.49%	5.02%
	Found	78.27%	6.03%	5.23%
im-PY-BPY-COF	Cacld.	82.99%	6.91%	4.60%
	Found	81.32%	6.56%	4.18%

Supplementary Table 4 Comparison of the ORR activity of *im*-PY-BPY-COF with various recently reported metal-free COFs catalysts in 0.1 M KOH aqueous solution.

Catalysts	$E_{1/2}$ (V vs. RHE)	E_O (V vs. RHE)	Tafel slope (mV dec^{-1})	TOF (s^{-1})	References
im-PY-BPY-COF	0.80	0.92	56.2	0.0170	This work
COF-JLU82	0.68	0.98	72.8	0.0044	S6
Azo-COF	0.68	0.88	89.0	0.0025	S7
JUC-528	0.70	0.83	65.9	0.0032	S8
TAB-HKH-COF-CNT	0.79	0.86	42.7	0.0013	S9
Oxazole-COF	0.75	0.85	79.0	0.0133	S10
JUC-606	0.70	/	59.9	0.0016	S11
JUC-616	0.78	1.02	52.9	0.0062	S12
JUC-610-CON	0.72	0.83	61.9	0.0035	S13
BTT-TAT-COF	0.77	0.87	71.0	0.0028	S14
JUC-607	0.72	0.85	61.0	0.0022	S15

Supplementary References

[S1] Kresse, G.; Furthmüller, J. *J. Comp Mater Sci.* **1996**, *6,* 15-50.

[S2] Kresse, G.; Furthmüller, J., *Phys. Rev. B.* **1996**, *54,* 11169-11186.

[S3] Perdew, J. P.; Burke, K.; Ernzerhof, M., *Phys. Rev. Lett.* **1996**, *77,* 3865-3868.

[S4] Kresse, G.; Joubert, D., *Phys. Rev. B.* **1999**, *59*, 1758-1775.

[S5] Monkhorst, H. J.; Pack, J. D., P*hys. Rev. B.* **1976**, *13*, 5188-5192.

[S6] Niu, W., Liu X. Stretchable Ionic Conductors for Soft Electronics. *Macromol. Rapid Comm.* **43**, 2200512 (2022).

[S7] Yan, X., Wang, B., Ren, J., Long, X., Yang, D. An Unsaturated Bond Strategy to Regulate Active Centers of Metal-Free Covalent Organic Frameworks for Efficient Oxygen Reduction. *Angew. Chem. Int. Ed.* **61**, e202209583 (2022).

[S8] Li, D. et al. Metal-Free Thiophene-Sulfur Covalent Organic Frameworks: Precise and Controllable Synthesis of Catalytic Active Sites for Oxygen Reduction. *J. Am. Chem. Soc.* **142**, 8104-8108 (2020).

[S9] Yang, S. et al. Interface engineering for modulating catalytic selectivity of covalent organic frameworks for oxygen reduction. *Mater. Today Chem.* **24**, 100936 (2022).

[S10] Li, X. et al. Catalytic Linkage Engineering of Covalent Organic Frameworks for the Oxygen Reduction Reaction. *Angew. Chem. Int. Ed.* **62**, e202304356 (2023).

[S11] Guan, X. et al. Chemically stable polyarylether-based covalent organic frameworks. *Nat. Chem.* **11**, 587-594 (2019).

[S12] Li, J. et al. Metal-free covalent organic frameworks containing precise heteroatoms for electrocatalytic oxygen reduction reaction. *J. Mater. Chem. A.* **11**, 18349-18355 (2023).

[S13] Chang, J. et al. Quasi-Three-Dimensional Cyclotriphosphazene-Based Covalent Organic Framework Nanosheet for Efficient Oxygen Reduction. *Nano-Micro Lett.* **15**, 159 (2023).

[S14] Jeon, J-P., Kim, YJ., Joo, SH., Noh, H-J., Kwak,SK., Baek, J-B. Benzotrithiophene-based Covalent Organic Framework Photocatalysts with Controlled Conjugation of Building Blocks for Charge Stabilization. *Angew. Chem. Int. Ed.* **62**, e202217416 (2023).

[S15] Chang, S., Li, C., Li, H., Zhu, L., Fang, Q. Stable Thiophene-sulfur Covalent Organic Frameworks for Oxygen Reduction Reaction(ORR). *Chem. Res. Chinese U.* **38**, 396-401 (2022).
