# Supplementary material for: Charging modulation of the pyridine nitrogen of covalent organic frameworks for promoting oxygen reduction reaction
Source: Nat Commun. 2024 Feb 29;15:1889. doi: 10.1038/s41467-024-46291-y (PMC10904383; doi:10.1038/s41467-024-46291-y)
Supplement: Supplementary file 3 — Description of Additional Supplementary Files [file 41467_2024_46291_MOESM3_ESM.pdf]

# Description of Additional Supplementary Files

**File Name:** Supplementary Data 1

**Description:** CIF files of PY-BPY-COF constructed using the Materials Studio software.
